# Central Nervous System Strongyloidiasis and Cryptococcosis in an HIV-Infected Patient Starting Antiretroviral Therapy

**DOI:** 10.1155/2012/575470

**Published:** 2012-08-08

**Authors:** Mónica Rodríguez, Paúl Flores, Víctor Ahumada, Lorena Vázquez-Vázquez, Claudia Alvarado-de la Barrera, Gustavo Reyes-Terán

**Affiliations:** ^1^Centro de Investigación en Enfermedades Infecciosas, Instituto Nacional de Enfermedades Respiratorias Ismael Cosío Villegas, Calzada de Tlalpan 4502, Colonia Sección XVI, Delegación Tlalpan, 14080 México, DF, Mexico; ^2^Departamento de Patología, Instituto Nacional de Enfermedades Respiratorias Ismael Cosío Villegas, Calzada de Tlalpan 4502, Colonia Sección XVI, Delegación Tlalpan, 14080 México, DF, Mexico

## Abstract

We report a case of *Strongyloides stercoralis* hyperinfection syndrome with central nervous system involvement, in a patient with late human immunodeficiency virus (HIV) infection starting antiretroviral therapy, in whom *Strongyloides stercoralis* larvae and *Cryptococcus neoformans* were isolated antemortem from cerebrospinal fluid. Our patient was not from an endemic region for the parasite, so strongyloidiasis was not originally suspected. For this reason, we conclude that *Strongyloides stercoralis* infection should be suspected in HIV-infected patients starting antiretroviral therapy in order to avoid potential fatal outcomes.

## 1. Introduction

Infection by *Strongyloides stercoralis* is endemic in tropical and subtropical regions of the world including Southern, Eastern, and Central Europe, Islands of the Caribbean, Latin America, Sub-Saharan Africa, and Southeast Asia. The number of people infected by this intestinal nematode is not well known, with estimates ranging from 30 to 100 million people living in 70 different countries [1]. In immunocompetent individuals, infection with *S. stercoralis* is usually asymptomatic, although pulmonary and gastrointestinal symptoms are common during acute and chronic infection. However, in immunocompromised patients, hyperinfection and dissemination of worms to ectopic sites (e.g., brain) causes severe illness. Both, hyperinfection and disseminated strongyloidiasis are more common in immunocompromised hosts [2]. Despite geographic overlaps, less than the expected number of cases of HIV-associated hyperinfection syndrome have been published [3–5]. Central nervous system (CNS) infection by *S. stercoralis* is uncommon, and the filariform larvae are found directly in the cerebrospinal fluid (CSF) rarely [6]. Here, we report a patient with late HIV-infection who had disseminated strongyloidiasis and cryptococcosis with CNS involvement soon after antiretroviral therapy initiation.

## 2. Case Report

A 30-year-old Mexican male patient was admitted to our institution due to a 3-week history of severe diarrhea, abdominal cramps, dry cough, progressive dyspnea, and fever. Patient informed consent for tests performed for clinical purposes using routine techniques was obtained on admission. His shortness of breath had progressed to resting dyspnea during the past 3 days. His vital signs were a heart rate of 112/min, blood pressure of 80/50 mm Hg, and respiratory rate of 38/min. He was febrile with a temperature of 38.7°C. Physical examination disclosed polypnea, central cyanosis, disorientation, psychomotor agitation with no meningeal signs, oropharyngeal candidiasis, absence of cervical adenopathy, tachycardia, fine rales throughout both lung fields, absence of genital visceromegalies or abdominal megalies, and a painless genital ulcer on the back of the penis. Blood gasometry values were pH 7.52; pCO_2_ 18; pO_2_ 41; and O_2_ saturation was 72% on room air. He had initiated combined antiretroviral therapy during the previous month, with a CD4 T-cell count of 145 cells/*μ*L and a viral load of 256 639 HIV RNA copies/mL (5.4 log). Ten years before he had travelled to endemic areas for *Strongyloides stercoralis* in Mexico.

**Figure 1:**
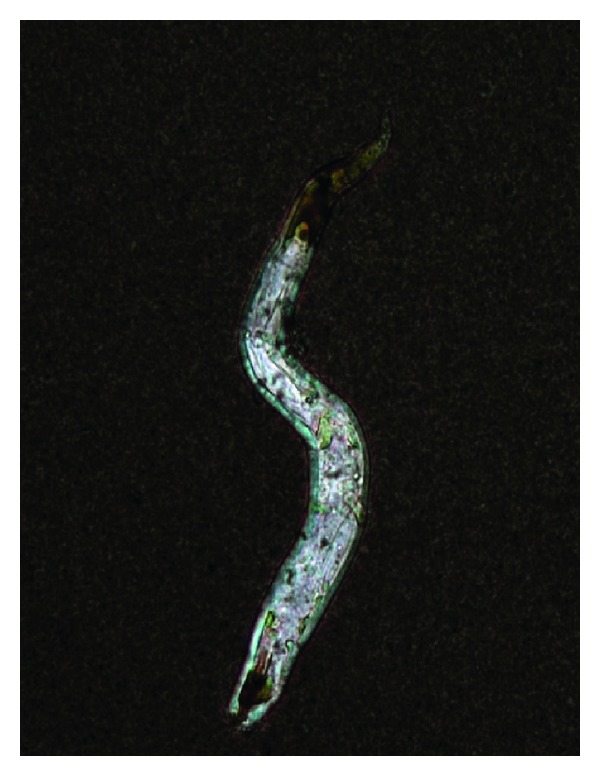
Rhabditiform larva of *Strongyloides stercoralis* recovered from cerebrospinal fluid sediment (India ink, x100).

Ten days after admission, generalized ground glass opacities were evident on chest X-ray, so the patient was immediately transferred to the intensive care unit (ICU) and he received mechanical ventilation. On ICU admission, he had a leukocyte count of 7600/*μ*L; a platelet count of 377 000/*μ*L; 89% neutrophils; 6% lymphocytes; 1% eosinophils; Hb level of 7.7 g/dL; Na level of 135 mEq/L; LDH level of 563 U/L; and ALP was of 544 IU/L. With the presumed diagnosis of *Pneumocystis carinii* pneumonia (PCP) and respiratory distress syndrome in an immunocompromised patient, treatment with TMP/SMX was initiated together with ceftriaxone, clarithromycine, and fluconazol. Prednisone 40 mg bid was administered via the enteral route for 5 days, followed by 40 mg once daily. His condition gradually deteriorated, with persistent fever and ventilatory function impairment. Cerebrospinal fluid collected through lumbar puncture was clear, slightly turbid, with an opening pressure of 27 cms H_2_O, with no cells, hyperproteinorraquia of 90 mg/dL, and glucose of 64 mg/dL. He had papilledema, and no focal neurological signs were detected. Cranial CT showed generalized cortical atrophy, unaltered ventricular size, and no structural lesions. Gram stain of CSF was negative, but India ink preparations revealed cryptococcal yeasts, and 2 *Strongyloides stercoralis* larvae were found in the cytological analysis (Figure 1). Therefore, administration of amphotericin B, flucytosine, ivermectin, and albendazol was initiated. *Strongyloides stercoralis* larvae were also found on bronchoalveolar lavage fluid cytology. Coproparasitoscopic and coprologic studies for *Mycobacterium tuberculosis* were negative, as well as CSF, blood and bronchoalveolar lavage cultures, and PCR. *Cryptococcus neoformans* was isolated latter from CSF, but it was not found in bronchoalveolar lavage nor in blood culture. The CD4 T-cell count was of 89 cells/*μ*L, and the viral load had decreased to 318 HIV RNA copies/mL (2.5 log). The patient developed multiple organ system failure and died 14 days after ICU admission.

## 3. Discussion

We have been able to identify only four cases in the literature of HIV-infected patients with disseminated strongyloidiasis and CNS involvement evidenced by direct finding of filariform larvae in CSF [6, 7], in meningeal vessels [8], or in meningeal spaces [9]. We report a case of *Strongyloides stercoralis* hyperinfection syndrome and disseminated cryptococcosis, in a patient with late HIV infection in whom *S. stercoralis* larvae and *Cryptococcus neoformans* were isolated antemortem from cerebrospinal fluid. Pulmonary and meningeal signs of parasite infection were predominant in our patient.

When the intact skin of a susceptible host interacts with the infective filariform larvae living in soil, transcutaneous infection occurs and the filariform larvae travel to the blood stream via subcutaneous lymphatics reaching the pulmonary circulation. Individuals infected by *S. stercoralis* may remain asymptomatic for long periods or they may course with acute or chronic nonspecific symptoms, but they can still develop mortal disease years after the exposure [10]. For this reason, diagnosis of strongyloidiasis relies on a high index of clinical suspicion, which is particularly difficult in those HIV-infected subjects who, as our patient, do not present eosinophilia, a common laboratory finding in immunocompetent population infected with this nematode [4]. In any case, the most important test for demonstrating *S. stercoralis* remains the repeated examination of stool over a number of consecutive days [11]. In our patient, direct examination of the parasite in CSF was conclusive for the diagnosis of strongyloidiasis.

Ivermectin 200 mg/kg/day is the treatment of choice for strongyloidiasis and, for patients with hyperinfection or disseminated infection, should be continued for at least 7–10 days or until resolution of symptoms [2]. However, deficient responses to conventional treatment have been reported in HIV-infected patients due to malabsorption and ileus [12] or to undefined causes [3]. Hence, a close followup and a more prolonged treatment with ivermectin or alternative agents (e.g., albendazol) have been considered in these cases. Our patient had CNS symptoms so the screening for opportunistic infections was done in CSF. We would like to emphasize that infection by *S. stercoralis* was then an incidental finding since Mexico City is not an endemic region for this parasite.

Here, we present a patient with infection by *Strongyloides stercoralis* and *Cryptococcus neoformans, *who had an apparent clinical deterioration soon after combined antiretroviral therapy initiation, and a 2.9 log decrease in viral load. Therefore, the clinical picture is consistent with an event of unmasking immune reconstitution inflammatory syndrome (IRIS). This condition occurs as a result of unmasking of clinically silent infection and is characterized by atypical exuberant inflammation and/or an accelerated clinical presentation, suggesting a restoration of antigen-specific immunity [13]. *Strongyloides stercoralis* might have been the main driver of IRIS [14], but we cannot exclude the contribution or perhaps the leading role of *Cryptococcus neoformans* in this episode [15]. In addition, the use of use of high doses of corticosteroids in patients with acquired immunodeficiency syndrome (AIDS) and cryptococcal meningitis has been associated to decreased survival. By consequence, the potential negative effects of prednisone in our patient should be considered.

We conclude that *Strongyloides stercoralis* infection should be suspected in HIV-infected patients starting antiretroviral therapy, even if they come from nonendemic areas, as subdiagnosis and therapeutic failure in this population may lead to fatal outcomes.
